# The ESC: The Dangerous By-Product of V(D)J Recombination

**DOI:** 10.3389/fimmu.2019.01572

**Published:** 2019-07-04

**Authors:** Alastair L. Smith, James N. F. Scott, Joan Boyes

**Affiliations:** School of Molecular and Cellular Biology, Faculty of Biological Sciences, University of Leeds, Leeds, United Kingdom

**Keywords:** V(D)J recombination, RAG proteins, genome instability, leukemia, double strand breaks

## Abstract

V(D)J recombination generates antigen receptor diversity by mixing and matching individual variable (V), diversity (D), and joining (J) gene segments. An obligate by-product of many of these reactions is the excised signal circle (ESC), generated by excision of the DNA from between the gene segments. Initially, the ESC was believed to be inert and formed to protect the genome from reactive broken DNA ends but more recent work suggests that the ESC poses a substantial threat to genome stability. Crucially, the recombinase re-binds to the ESC, which can result in it being re-integrated back into the genome, to cause potentially oncogenic insertion events. In addition, very recently, the ESC/recombinase complex was found to catalyze breaks at recombination signal sequences (RSSs) throughout the genome, via a “cut-and-run” mechanism. Remarkably, the ESC/recombinase complex triggers these breaks at key leukemia driver genes, implying that this reaction could be a significant cause of lymphocyte genome instability. Here, we explore these alternate pathways and discuss their relative dangers to lymphocyte genome stability.

## Introduction

V(D)J recombination is essential to generate a diverse adaptive immune system that can respond to vast numbers of potential pathogens. This is achieved, in part, from the unique arrangement of antigen receptor loci where multiple copies of variable (V), diversity (D), and joining (J) gene segments lie upstream of constant exon(s). During V(D)J recombination, one of each V, D (if present), and J gene segments are somatically recombined at random to generate the variable exon of the antigen receptor. The stochastic selection of gene segments, along with their imprecise joining enables production of a highly diverse antigen receptor repertoire (1).

The products of the lymphoid specific recombination activating genes 1 and 2 (*RAG1* and *RAG2*) (2), are essential for V(D)J recombination (3, 4). These proteins recognize recombination signal sequences (RSSs) that flank each V, D, and J gene segment and consist of a conserved heptamer (CACAGTG) and nonamer (ACAAAAACC), separated by non-conserved “spacers” of either 12 ± 1 or 23 ± 1 bp. Importantly, efficient recombination only occurs between RSSs with dissimilar spacers, the “12/23 rule” (5).

**Figure 1:**
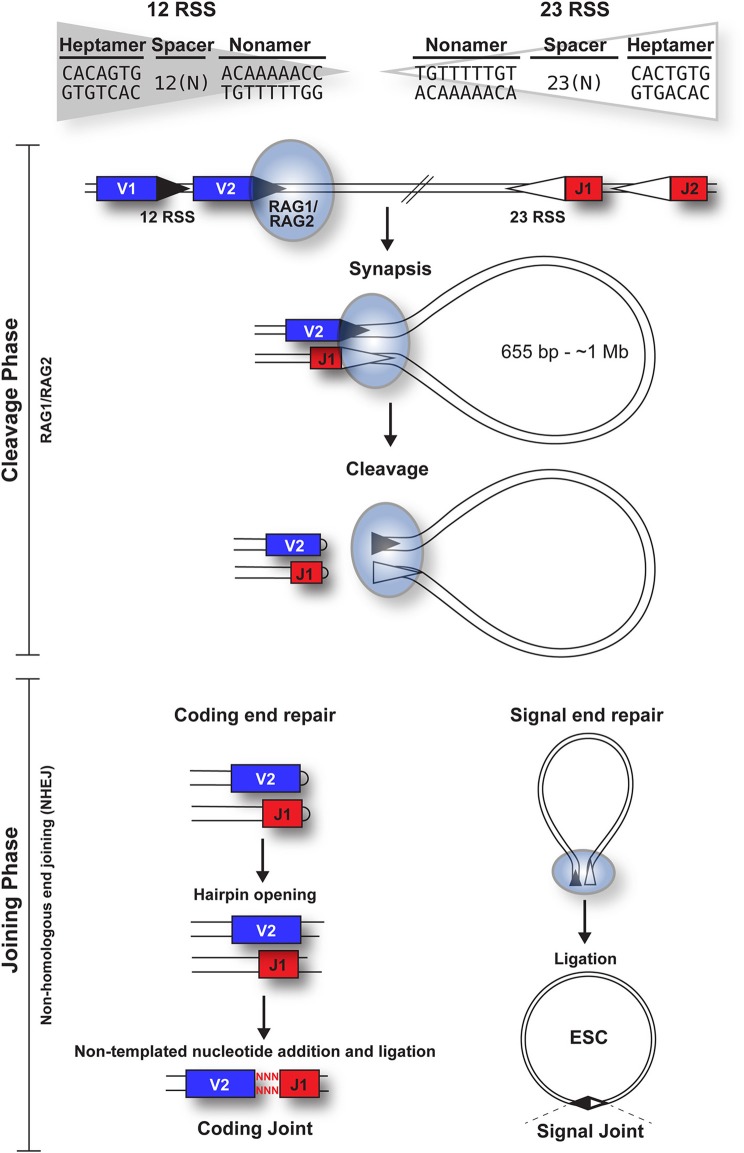
Overview of the cleavage and joining phases of V(D)J recombination. The cleavage phase begins when a RAG1 + RAG2 hetero-tetrameric complex (blue oval) binds to either a 12-RSS (filled triangle) or 23-RSS (open triangle), with the consensus sequences shown. This complex then synapses with a partner RSS of the complementary spacer length. The RAG complex subsequently nicks one DNA strand of each RSS and catalyzes the hydrophilic attack by the resulting hydroxyl group onto the other DNA strand in a direct *trans*-esterification reaction, to generate hairpinned coding ends and blunt signal ends. Both the coding and signal ends are repaired by the NHEJ machinery, although coding ends are often imprecisely repaired to increase diversity, as indicated by red nuclotides. The signal ends remain bound to the RAG proteins until *RAG* expression is downregulated; subsequent ligation of the signal ends forms a signal joint (SJ) and results in the generation of an episomal circle, the excised signal circle (ESC). In humans, these range from 655 bp (at the *TCRB* locus) to ~1 Mb (at the *IgH* locus).

V(D)J recombination can be divided into cleavage and joining phases [Figure 1; (6)]. Cleavage is initiated when a hetero-tetrameric complex of RAG1 and RAG2 assembles on either a 12- or 23-RSS (7) and subsequently captures a complementary RSS. Upon formation of a stable synaptic complex, the DNA is unwound at the 5' end of the heptamer (8), followed by introduction of a single-strand DNA nick at the heptamer-coding sequence boundary by the DDE catalytic motif of RAG1 (9–11). The exposed free 3' hydroxyl group then attacks the opposite DNA strand in a direct *trans*-esterification reaction (2), yielding a pair of covalently sealed hairpins at the coding ends, and blunt signal ends (1).

Repair of the four broken DNA ends is achieved by the non-homologous end joining (NHEJ) machinery (12). Whilst the coding ends undergo extensive processing, resulting in addition or deletion of bases to increase antigen receptor gene diversity, the RSSs at the signal ends are precisely joined in a head-to-head arrangement, generating a signal joint (SJ). Usually, V(D)J recombination deletes the DNA between the gene segments, to generate an excised signal circle [ESC; (1)] that is covalently sealed at the SJ (Figure 1). However, recombination, primarily of the *Ig*κ locus, can result in inversion of the intervening DNA and retention of the SJ in the genome. Notably, production of every functional antigen receptor gene generates at least one, and up to 10 ESCs, depending on the level of non-productive rearrangement.

The ESC is a non-replicative episome which is likely lost during cell division. Nonetheless, it persists in chicken T cells for approximately 2 weeks (13) and in primates, this appears to be substantially longer (14). Little to no ESC degradation has been observed and both T cell receptor excision circles (TRECs) and KRECs, generated by recombination to the kappa deleting element during allelic and isotypic exclusion, have proved to be excellent markers of recently generated T- and B-cells, respectively, with 70% of newly produced T cells and 50% of transitional and naïve B cells testing positive (15, 16).

Due to their lack of coding capacity and eventual loss from the cell, it is reasonable to ask why cells bother to generate ESCs. One possibility is that SJ formation is required to maintain chromosome integrity during inversional recombination and this mechanism is simply retained during deletional recombination. Generation of a circle during deletional recombination has the further advantage of sequestering potentially reactive DNA ends. Recent studies strongly suggest, however, that far from being inert, ESCs are crucial constituents of reactions that have potentially devastating consequences for lymphocyte genome stability.

## RAG Mediated Signal End Transposition

The first inkling that the ESC poses a potential threat stemmed from the remarkable similarity between the core domain of RAG1, including the catalytic DDE motif, and the *Transib* family of transposes (17). Furthermore, ProtoRAG, a recently discovered transposon in lancelets, is comprised of both RAG1- and RAG2-like genes, flanked by terminal inverted repeats (TIRs), similar to RSSs. This strongly implies that the RAG recombinase evolved from an ancient transposase that was acquired into the jawed vertebrate genome by horizontal gene transfer (18).

Transposases recognize short sequences surrounding the transposon and introduce double strand breaks (DSBs) between these recognition sequences and the flanking chromosomal DNA, in much the same way as the V(D)J recombinase recognizes RSSs (19). The initial breakage of DNA at the antigen receptor loci and the hairpin end structures formed is also analogous to the process by which hAT family transposons are excised from their host genome (20). Furthermore, RSSs resemble inverted repeats found at either end of a transposon and RAG proteins remain associated with RSSs after DNA cleavage, which is also common in transposition reactions (21). Consequently, it was not surprising that RAG proteins were found to mediate signal end transposition, at least *in vitro*.

**Figure 2:**
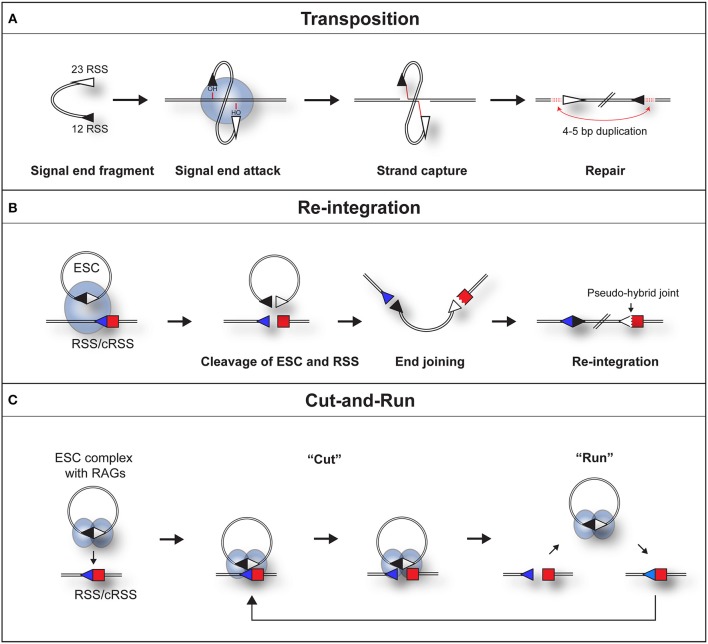
Three pathways by which a RAG/SJ complex can lead to genomic instability. **(A)** During RAG mediated signal end transposition, the free hydroxyl groups of the signal ends are used by the RAG proteins (blue oval) in a direct trans-esterification reaction to attack target DNA. DNA polymerase repairs the gaps that are generated, to produce a 4–5 bp repeat of DNA flanking the insertion (red dotted lines). **(B)** Re-integration of an ESC into a genomic RSS. The SJ forms a synaptic complex with a genomic RSS (blue triangle). RAG proteins cleave both the SJ and RSS in a *trans*-V(D)J recombination reaction. End repair by the non-homologous end joining machinery results in the formation of a chromosomal signal joint and a pseudo-hybrid joint, which typically has imprecise end processing. **(C)** Cut-and-run. The SJ forms a synaptic complex with a genomic RSS (blue triangle). This results in cutting of the genomic RSS but *not* the SJ, most likely because RAG complexes (blue ovals) bind to each RSS of the SJ and block its cutting. The cleaved RSS is released from the SJ/RSS complex, but the RAG/ESC complex remains intact to potentially generate further DSBs at other genomic RSSs. In some instances, it is possible that RAGs nick the SJ which may make it easier for the RAG-SJ complex to capture a partner RSS. Since consensus RSSs were used in the published work (22), the cut-and-run mechanism could potentially occur with ESCs from all antigen receptor loci, although the frequency of consensus RSSs is higher at immunoglobulin loci.

Indeed, both the Gellert and Schatz laboratories demonstrated that in the presence of RAG proteins, DNA substrates flanked by a pair of dissimilar RSSs undergo an intermolecular transposition reaction into DNA targets [Figure 2A; (23, 24)]. Furthermore, the SJs were shown to be opened by a “nick-nick” reaction, whereby RAGs sequentially nick at the heptamer-heptamer junction on each strand, to generate the OH^−^ groups required to attack the target DNA during transposition (25).

Despite clear evidence for RAG-mediated transposition events *in vitro*, the impact of these events on genomic stability appears inconsequential. Only two natural integration events have been described (26); in each case, an excised signal end fragment from the *TCR*α locus was integrated into the *HPRT* (hypoxanthine phosphoribosyltransferase) locus in human T cells. Although a handful of transposition events have been observed experimentally, characterized by a 5 bp duplication at the insertion site (27), no single documented case of leukemia or lymphoma can be traced to a transposition event. The most likely explanation is the suppression of transposition by the RAG2 C-terminus (28): Full length RAG2 does not support efficient transposition (29), in contrast to “core” RAG2 (amino acids 1–387), commonly used *in vitro*. This suppression appears to be achieved by the RAG2 C-terminal domain stabilizing the cleaved signal complex (30), to prevent subsequent capture and transposition of signal ends. Consequently, transposition reactions are almost completely suppressed *in vivo*.

## RAG Mediated ESC Re-integration

Although signal ends are not efficiently transposed into the genome, ESCs can be readily cleaved (25) and re-bound by RAG proteins (31) *in vivo*. As a potential consequence, the ESC has been found to undergo relatively efficient re-integration into the genome at RSSs and cryptic RSSs (cRSSs), i.e., RSSs outside the antigen receptor loci that resemble consensus RSSs, via a *trans*-V(D)J recombination reaction. Indeed, in an *ex vivo* assay, Nadel et al. observed that RAGs can integrate genuine SJ sequences into 12-RSS target substrates via synapsis with the 23-RSS of the SJ (32). This generates a new SJ and a pseudo-hybrid joint, where the RSS is joined to a coding end (Figure 2B). Consistent with *bona fide* trans-V(D)J recombination, the re-integration reaction usually does not violate the 12/23 rule (32) and depends on Artemis to open the hairpin at the coding end. Furthermore, pseudo-hybrid joints, with characteristic processing of the coding end, are observed in mouse thymocytes (32).

A genome wide screen of signal end sequences in precursor T-cells, similarly confirmed the presence of ESC insertions *in vivo*. Approximately half were due to re-integration of an ESC, with a distinct absence of transposition events (33), suggesting that re-integration is the principal mechanism of ESC insertion. A high rate of ESC re-integration was also observed using episomes carrying *LMO2* and *TAL2* cRSSs (32), implying that re-integration at proto-oncogenes is feasible. Given that ESCs are highly likely to carry promoters, such as those adjacent to V gene segments, such re-integration could upregulate oncogenes, contributing to malignant transformation. Notably, the RAG2 C-terminus also suppresses ESC re-integration by about 7-fold (33). This may relate to the degradation of RAG2 outside of G1 by phosphorylation of threonine 490 (34), which has been shown to suppress genome instability (35). Nevertheless, based on the experiments described above, ESC re-integration has been estimated to occur in 1 in 1,000,000 to 1 in 10,000 thymocytes (32, 33). Given that millions of lymphocytes are generated each day, this is equivalent to ~5,000 re-integration events per genome per day (32).

Despite the high estimated number of re-integration events, none has been unequivocally linked to malignant transformation. Moreover, only one natural re-integration has been reported, where a *TCR*α-derived ESC was inserted into a cRSS in the *HPRT* locus (36). The large discrepancy between the estimated re-integration frequency and actual carcinogenic events begs the question of why more disease-causing re-integrations are not observed. One possibility is that re-integration results in B- or T- cell death, either by insertional mutagenesis into critical genes or because re-integration of an ESC, which can be up to 1 Mb in humans, is error-prone and generates DSBs that trigger apoptosis via the p53 surveillance pathway. A second possibility is that re-insertion occurs, but is not detected (32). Since recombination is stochastic, it is difficult to predict which ESCs will be generated, and moreover, the ESC could be potentially inserted at any of 10 million cRSSs (37), which makes screening for reintegration difficult. Indeed, over a decade after re-integration was first described, its contribution to lymphoid malignancies remains unknown.

Although not strictly involving ESCs, the Robbiani laboratory recently described a related mechanism of RAG-mediated genome instability (38). Using bespoke translocation capture (TC-seq) and insertion capture sequencing (IC-seq), they found that RAGs can release DNA fragments (with signal ends, coding ends or hybrid ends) from antigen receptor loci, independent of normal recombination, and these fragments reintegrate into a RAG-independent DSB elsewhere in the genome. By developing a novel pipeline to analyse whole genome sequencing data, they found 5 out of 34 acute lymphoblastic leukemia (ALL) and follicular lymphoma patients displayed genomic insertions from the antigen receptor loci. However, the authors believe this is an underestimate due to limitations in sequencing depth and read lengths. Notably, with such improvements in whole-genome sequencing, this analysis pipeline could be used additionally to detect RAG-mediated ESC re-integration in lymphoid malignancies.

## Cut-and-Run

Whilst re-integration events clearly occur, it was shown recently that synaptic complex formation between an ESC and RSS *in vitro* results in efficient RSS cleavage, whereas the ESC itself is barely cut (22). This asymmetric cleavage of an ESC/RSS complex does not appear to be an *in vitro* artifact as assays using a cell line derived from an ALL patient, showed that episomes carrying a 12- or 23-RSS are also cleaved readily whereas substrates bearing a SJ (ESC) exhibit negligible cutting (22).

This phenomenon can be best explained by RAGs binding to both RSSs of the ESC (22), thereby occluding the heptamer-heptamer boundary of the SJ, preventing its cleavage. What makes this reaction particularly dangerous is following cleavage, the broken RSS is released, generating a DSB (22) whereas the ESC/RAG complex remains intact. Although not yet observed directly, hypothetically, the RAG/ESC complex could catalyze further breaks at new cRSSs in a reaction termed “cut-and-run” (Figure 2C). This reaction could potentially continue until the RAG proteins are downregulated and/or the ESC is eventually cleaved—with disastrous consequences for the lymphocyte genome. Indeed, analysis of chromosome breakpoints caused by the RAG/ESC complex, using linear amplification-mediated, high-throughput genome-wide sequencing [LAM-HTGTS; (39)] showed a significant overlap between cut-and-run-mediated breakpoints and those observed in *ETV6/RUNX1*-positive ALL patients (22, 40). Moreover, breakpoints in eight out of the eleven most commonly mutated genes in B-cell ALL were observed in the presence of the ESC (22). This strongly implies that cut-and-run has a role in the development of *ETV6/RUNX1*-positive ALL, and potentially other lymphoid cancers.

## The Dangers of Cut-and-Run Compared to Re-integration

The discovery of two distinct mechanisms by which the ESC triggers genome instability raises the fundamental question of which poses the greater danger. This will be influenced by both the reaction frequency and damage caused by each reaction.

### Reaction Frequency

The overall reaction frequency will depend on its actual frequency as well as the availability of reaction components. Both re-integration and cut-and-run require a complex between RAGs and the ESC and thus will be restricted to cells where both are present, such as pro- and pre-B cells as well as immature B cells, where RAGs are upregulated for receptor editing and receptor revision (41, 42). Moreover, whilst re-integration can theoretically occur with either a covalently closed ESC or open SJ, cut-and-run requires the ESC to be covalently closed. Notably, SJs remain unligated following recombination until RAGs are down-regulated, either as a result of cell replication (34, 43) or following productive antigen receptor recombination (44). This will therefore further restrict cut-and-run; nonetheless, it could occur in normal lymphocytes, for example, by using ESCs in pre-B cells that were generated by IgH recombination in pro-B cells. Substrates for both reactions are likely to be substantially increased, however, in cancer cells. Indeed, a number of pre-leukaemic (45) and leukaemic cells (46) continually express RAGs, triggering ongoing recombination and increased production of ESCs (47). Because these cells continually divide, the generation of covalently closed ESCs is likely to be particularly high, thereby enhancing the risks of further genome instability.

Nonetheless, the window in which cut-and-run or re-integration can occur will be restricted by the short half-life of RAG1 [~15–30 min; (48, 49)] and by the cell-cycle dependent degradation of RAG2 outside of G1 (34, 35). Not only this, but it appears that the lymphocyte genome has tried to protect itself against off-target RAG cleavage: Only ~3500 of the millions of cRSSs are occupied by RAG1 (50), substantially limiting where breaks could occur. In addition, genomic regions outside of the antigen receptor loci that are enriched for RAG binding were found to be depleted of RSSs, a mechanism suggested to protect active transcriptional start sites from off-target RAG cleavage (50).

Yet further restrictions on RSS cleavage are imposed by local chromatin modifications. Indeed, RSSs need to be accessible to RAGs and to have proximal nucleosomes marked by acetylation of lysine 27 of H3 (H3K27Ac) for RAG1 binding (51) and trimethylation of lysine 4 of H3 (H3K4me3) for RAG2 (52, 53). Whilst these factors undoubtedly provide some protection, cleavage at cRSSs clearly still occurs. Indeed, breaks at cRSSs in cancer driver genes were found to be a predominant cause for cancer progression in *ETV6/RUNX1* ALL, and our LAM-HTGTS experiments suggest the ESC could play a role in causing some of these breaks (22, 40).

Interaction of RAG2 with H3K4me3 via its PHD finger has a further regulatory role, namely to overcome the auto-inhibition of RAG1 cleavage, imposed by RAG2 (54–56). Since the chromatin modifications present on ESCs are currently unknown, it is difficult to determine if the ESC/RAG complex is affected by altered H3K4me3 levels. Nonetheless, formation of the RAG/ESC complex generates half of the synaptic complex required for either cut-and-run or re-integration, meaning that these reactions require just one accessible cRSS in the genome (22). This, together with the intrinsic mobility of the RAG/ESC complex, is expected to increase the chances of the RAG/ESC complex “finding” genomic (c)RSSs with the correct chromatin modifications. Furthermore, RAG proteins have been shown to nick the ESC *in vitro* and *in vivo* (25), which could also increase the chances of the RAG/ESC complex capturing a partner RSS, although the level of ESC nicking that we observed was noticeably lower than at an RSS (22).

Considering the reaction rate, cut-and-run is far more likely to occur. In fact, *in vitro* reactions suggest there is a 10-fold greater likelihood of cutting just at the RSS (for cut-and-run) compared to cutting at both the RSS and ESC (22), to enable re-integration. Furthermore, the fact that the ESC/recombinase complex remains intact following cutting at one site (22) means that cut-and-run could potentially trigger a series of DSBs, resulting in significant damage. By contrast, re-integration if it occurs, is very likely to occur only once as the event typically mutates one of the RSSs (32), preventing further reactivity. Whilst it is difficult to know the actual frequency of cut-and-run, using γH2AX as a marker for DSB, physiological levels of RAGs (57) and close to physiological levels of ESCs (3–6 ESCs per cell), an average of two additional DSBs were observed in ~20% of cells in the presence of the ESC, compared to control cells (22). This is considerably higher than the estimates for re-integration of 1 in 100,000 cells (33), suggesting that cut-and-run could be both frequent and dangerous.

### Damage Caused by Cut-and-Run or Re-integration

Both re-integration of the ESC and the cut-and-run reaction pose significant threats to genomic stability but theoretically, a single ESC re-integration event has the potential to cause a greater degree of genomic disruption due to the insertion of up to 1 Mb of DNA. Furthermore, due to the presence of strong promoters within the antigen receptor loci, re-integration of an ESC could upregulate genes next to the insertion site, including proto-oncogenes such as *LMO2*. Not only this, but Nadel et al. observed two cases of translocations which appeared to be associated with insertion events (32), suggesting secondary re-arrangement events may occur after integration.

A single cut-and-run event has, in theory, a lower probability of genomic disruption compared to ESC re-integration. However, the damage caused will depend entirely on the processing of the break. Asymmetric ESC/RSS cleavage generates one hairpinned and one blunt end (22); crucially, these broken ends are released and are not chaperoned by RAGs to the NHEJ machinery (58). Although it is possible that the NHEJ proteins are recruited independently, resulting in hairpin opening and repaired DNA that closely resembles the sequence prior to cleavage [akin to open and shut joints; (59)], it is also possible that the released ends are processed by the error-prone alternative NHEJ pathway or that they may be used as substrates for chromosome translocations. Indeed, DSBs generated by cut-and-run were found to readily undergo translocation, as determined by the LAM-HTGTS assay (22, 39). Moreover, one of the most common V(D)J recombination errors is the end donation reaction, where a broken RSS becomes joined with an independently broken DNA end (19). It is possible that some of the breaks generated by cut-and-run are substrates for such reactions.

### Relationship to Development of Cancer

The relative dangers of these reactions can be further estimated by considering their links to cancer. To date, no documented examples exist of cancers triggered by re-integration. This may be because re-integration is hard to detect and it was argued that since approximately one third of T-ALL cases have oncogene activation without abnormal karyotypes (32), oncogenesis triggered by pathways such as re-integration may be involved. However, many whole genome sequences from patients have since become available, which show relatively small chromosome changes that would not be detected karyotypically but nonetheless lead to oncogene activation. Crucially, many of these small changes in *ETV6/RUNX1*-positive ALL occur at RSSs (40) and there is a strong correlation between the breaks in patients and those caused by cut-and-run (22, 40). Furthermore, about half of the chromosome abnormalities mapped via whole genome sequencing show breaks at just one RSS (40). It is difficult to account for such breaks during normal V(D)J recombination given the stringency for coupled RSS cleavage and end joining. Cut-and-run could account for such breaks.

## Concluding Statement

It is clear that the ESC is far from inert but instead poses a significant threat to genome stability. Although re-integration has the potential for significantly greater damage, its low frequency compared to cut-and-run and the low probability of integrating at a proto-oncogene significantly reduces its overall danger. Cut-and-run, on the other hand, appears to be frequent and could be a source of breaks for the major chromosome alterations associated with errors in V(D)J recombination. However, to fully understand the impact of cut-and-run, further experiments are required to determine (a) the outcomes of the released broken ends, (b) if the ESC “runs” to trigger subsequent genomic breaks, and (c) if cut-and-run contributes to other B and T cell cancers.

## Author Contributions

All authors listed have made a substantial, direct and intellectual contribution to the work, and approved it for publication.

### Conflict of Interest Statement

The authors declare that the research was conducted in the absence of any commercial or financial relationships that could be construed as a potential conflict of interest.
